# Beyond Self-Report: Addressing Suicide Risk in Personal Narrative Crisis

**DOI:** 10.1192/j.eurpsy.2025.2361

**Published:** 2025-08-26

**Authors:** I. M. Lopes, D. Seabra, G. Santos, N. Ramalho, T. Coelho Rocha, J. Alves Leal, J. F. Cunha, J. Moura, D. Santos, A. Garcia, M. Rosa, C. Pires

**Affiliations:** 1 Psychiatry, ULS Arco Ribeirinho; 2 Psychiatry, USL Arco Ribeirinho; 3 Psychiatry, ULS Barreiro, Barreiro, Portugal

## Abstract

**Introduction:**

Effective suicide risk assessment remains a significant challenge in psychiatric care, particularly when dealing with patients undergoing acute crises. This case study highlights the limitations of current assessment practices and underscores the need for a more nuanced understanding of suicide risk, particularly through the lens of personal narrative crisis and Suicide Crisis Syndrome (SCS).

**Objectives:**

The objective of this study is to describe a clinical case involving a patient with Suicide Crisis Syndrome (SCS), which emerged from a fundamental alteration in his personal life narrative. Additionally, this study reflects on the existing clinical gap due to the lack of consensual or homogeneous approaches for detecting suicide risk in psychiatric patients experiencing a crisis.

**Methods:**

**Methodology:** A detailed review of the clinical process and hetero-anamnesis was conducted. The case explores the concept of SCS—an acute mental state that precedes a genuine suicide attempt.

**Results:**

The patient, a 45-year-old single male with no children, had a biographical history marked by traumatic events during military service and the loss of a child in combat at age 30. For years, he exhibited symptoms consistent with Post-Traumatic Stress Disorder (PTSD), which he managed effectively through meditation and martial arts, resulting in total symptom remission for over a decade. However, following complex cardiac surgery two years ago, he experienced a significant loss of functionality and autonomy, leading to the abandonment of his martial arts practice and lifelong profession as a rehabilitation therapist. This change had profound emotional, behavioral, and socio-economic impacts, resulting in depressive symptoms. During psychiatric evaluation, the patient exhibited affective dysregulation, marked hopelessness, a sense of loss of meaning, feelings of non-belonging, social defeat, and difficulty adjusting unrealistic goals to his current situation. Although he denied active suicidal ideation, he reported intrusive thoughts of death.

**Conclusions:**

**Conclusion and Implications:** The assessment of self-reported suicidal ideation is often unreliable. There is an urgent need to adopt more comprehensive approaches that focus on the personal narrative crisis and SCS, as current evidence suggests that SCS is a strong predictor of actual suicidal behavior within 1-2 months after discharge.

**Disclosure of Interest:**

None Declared

